# Impact of *TCF4* Repeat Number on Resolution of Corneal Edema after Descemet’s Stripping Only in Fuchs Dystrophy: A Pilot Study

**DOI:** 10.3390/vision5040047

**Published:** 2021-10-09

**Authors:** Natasha Spiteri, Nino Hirnschall, Katherine van Bysterveldt, Alec Lin Hou, Gregory Moloney, Matthew Ball, Andrea L. Vincent

**Affiliations:** 1 Department of Ophthalmology, Countess of Chester Hospital, Liverpool Road, Chester, Cheshire CH2 1UL, UK; 2 VIROS-Vienna Institute for Research in Ocular Surgery, a Karl Landsteiner Institute, Hanusch Hospital, Heinrich-Collin-Straße 30, 1140 Vienna, Austria; nino@hirnschall.at; 3 Department of Ophthalmology, Faculty of Medical and Health Science, New Zealand National Eye Centre, University of Auckland, Auckland 1142, New Zealand; k.vanbysterveldt@auckland.ac.nz (K.v.B.); a.hou@auckland.ac.nz (A.L.H.); a.vincent@auckland.ac.nz (A.L.V.); 4 Sydney Eye Hospital, 8, Macquarie Street, Sydney, NSW 2000, Australia; gregorymoloney@yahoo.com.au (G.M.); mattball22@yahoo.com.au (M.B.); 5 Discipline of Ophthalmology, Sydney Medical School, The University of Sydney, Sydney, NSW 2006, Australia; 6 Eye Department, Greenlane Clinical Centre, Auckland District Health Board, Auckland 1051, New Zealand

**Keywords:** descemetorhexis, descemet stripping only (DSO), fuchs endothelial corneal dystrophy, *TCF4* repeat

## Abstract

Purpose: To investigate whether Fuchs endothelial corneal dystrophy (FECD) genotype, specifically transcription factor 4 (*TCF4)* CTG triplet repeat “load” predicts time to clearance following Descemet’s Stripping Only (DSO). Methods: This prospective, interventional trial was conducted on consecutive FECD patients undergoing DSO. Genetic analysis using patients’ saliva was performed to assess the extent of CTG expansion using short tandem repeat analysis, corroborated gel electrophoresis and Sanger sequencing. Polymerase chain reaction and bidirectional Sanger sequencing was undertaken. Partial least square regression and logistic regression modelling was used to evaluate the predictive power of *TCF4* repeats on corneal clearance. Results: Of 11 eyes of 11 patients, 8 showed complete corneal clearance. For these 8 patients, mean *TCF4* allele repeat was 24.8 (SD: 23.7, range: 11–63) and 63.4 (SD: 30.3; range: 11–97), respectively. In total, 9/11 (81.8%) had expanded CTG repeats (>40) in one allele. In cases with an allele repeat ≥80, there was a significantly increased risk of corneal non-clearance (odds ratio 18.2, *p* = 0.009). Conclusion: Whilst it was not possible to predict time to corneal clearance based on CTG repeats, there is a significant correlation between allele repeats and achievement of corneal clearance.

## 1. Introduction

Fuchs endothelial corneal dystrophy (FECD) with corneal decompensation is one of the commonest indications for corneal transplant surgery, but alternative surgical techniques are currently being explored, such as planned descemetorhexis (peeling the Descemet membrane) without endothelial keratoplasty [1,2,3,4,5,6]. This technique obviates the need for donor corneal tissue. The process of corneal clearance following Descemet’s stripping is potentially lengthy and sometimes fails, requiring subsequent keratoplasty. Patient classification into “fast”, “slow” and “non-responders” has been proposed to describe this variability [4]. It is therefore becoming increasingly necessary to define which patients with FECD require corneal transplantation and which do not, with the ultimate goal being to decrease the burden on eye bank corneal donor tissue supply.

For this study, we hypothesized the underlying FECD genotype may influence response to Descemet’s stripping. Specifically, whether the transcription factor 4 (*TCF4)* CTG triplet repeat “load” would predict time to healing following Descemet’s Stripping Only (DSO).

## 2. Materials and Methods

Subjects were recruited in a prospective, consecutive manner following referral to a single tertiary center for treatment of FECD. Genetic analysis was performed masked to subjects’ condition and postoperative outcome, at a separate institution.

The study was performed in accordance with the Declaration of Helsinki, and received approval from the local research ethics committee both at the surgical site (South Eastern Sydney Area Health Service HREC 14.238) and the genetic testing facility (Health and Disability Ethics Committee, New Zealand NTX/06/12/161). All subjects received written and verbal information prior to informed consent and enrollment.

Subjects underwent DSO with removal of the 4.0 central affected endothelium by two surgeons (G.M. and M.B.) using a reverse Sinskey hook [7]. Postoperatively, subjects were treated with topical chloramphenicol 0.5%, ketorolac 0.5% and sodium chloride 5%, all four times per day until corneal clearance. Subjects were seen at post-operative day 1, week 1 and then monthly thereafter and clinically examined for clearance of corneal edema with slit-lamp biomicroscopy. Non-clearance was defined as failure of formation of a clear zone between descemetorhexis margin and edematous area, or halted progression of clearance over 3 consecutive study visits. Subjects who had non-clearance of edema either had off-label rescue treatment with topical ripasudil or underwent endothelial keratoplasty, with outcomes previously published [7]. These subjects were included in the analysis as “non-clearance” in an intention to treat fashion.

### 2.1. Molecular Analysis

Biological specimens were obtained by saliva collection (Oragene, DNAGenotek, Ottawa, ON, Canada), and DNA extraction followed manufacturer’s instructions. The extent of *TCF4* expansion was measured using short tandem repeat (STR) analysis (Primers described in Wieben et al. [8], and corroborated gel electrophoresis and Sanger sequencing. An unaffected control was analyzed to ensure validity. STR results were analyzed with Strand software (Strand Life Sciences, Bengaluru, India).

### 2.2. Statistical Analysis

In accordance with previous studies [9] *TCF4* CTG triplet repeats of ≥40 were considered as an expanded allele and <40 as a non-expanded allele, and this was used to categorize subjects into the following sets of groups for statistical comparison:

Group 1: If both *TCF4* alleles ≤ 40 CTG repeats;
Group 2: If one *TCF4* allele > 40 CTG repeats;
Group 3: If both *TCF4* alleles > 40 CTG repeats.

All the descriptive analysis was conducted using STATA 14 software (STATA Corp). The Fisher’s exact test was used to compare binary outcomes (clearance yes/no). A *p*-value of <0.05 was considered statistically significant. To evaluate the predictive power of explanatory variables for corneal clearance time, a partial least square regression (PLSR) model was used with data analysis software (Xlstat 2012; New York, Addinsoft, USA). For binary outcome parameters (corneal clearance achieved: yes/no) a logistic regression model was used. The main outcome of both regression models is the variable importance for projection (VIP), representing the predictive power of the regression model. A value above 0.8 is considered to represent a high predictive power. The liability of the PLSR model was evaluated using a boot strapping model [10].

## 3. Results

### 3.1. Baseline Characteristics

All 11 subjects completed follow-up to the end of the study period and were included in the analysis. Mean age was 64.3 years (SD: 10.2; range: 39–84). All patients were female. 

Out of 11 eyes of 11 patients, 8 eyes showed corneal clearance during the duration of the study. Mean time to clearance was 3.5 months (SD: 1.6, range: 2.0 to 6.0). Mean allele repeat was 24.8 (SD: 23.7, range: 11–63) and 63.4 (SD: 30.3; range: 11–97), respectively. Concerning pre-operative corneal parameters, endothelial cell count (measured superiorly) was on average 2064.9 cells/mm^2^ (SD: 671.8; range: 850 to 3146) and central corneal thickness was on average 618.4 µm (SD: 49.0; range: 534 to 699). A full list of demographics and pre-operative phenotypes, number of *TCF4* trinucleotide repeats and the time to clearance of edema for each eye studied can be found in Table 1.

9/11 (81.8%) individuals had expanded CTG repeats (>40) in at least one allele.

### 3.2. Corneal Clearance

There was no statistically significant difference in time to clearance between groups 1, 2 and 3, respectively (*p* = 0.19). Similarly, there was no significant difference in clearance versus non-clearance amongst these three groups (*p* = 0.33). When comparing the predictive power of corneal clearance time of the different parameters, age and the *TCF4* repeat were found to have a relevant influence (Table 2).

In three of the 11 eyes, no corneal clearance was achieved within the study duration. The *TCF4* repeat was found to have a highly relevant influence on the clearing probability. 

Considering a high allele repeat as a risk factor, the odds ratio was calculated in the next step and it was shown that an allele repeat of at least 80 lead to a significant increase in risk:

Highest allele repeat ≥80: odds ratio 18.2 (*p* = 0.009).

## 4. Discussion

This study shows that a high repeat in *TCF4* is a significant risk factor for incomplete clearance after DSO. To our knowledge it is the first study of its kind. It was not possible to predict time to clearance in successful cases based on CTG triplet repeat load in the *TCF4* gene (although there is a trend that allele repeats correlate with corneal clearance time). 

The effect of *TCF4* and FECD was intensively investigated in previous studies [8,11]. In patients with FECD, expansion of the *TCF4* CTG triplet greater than 30–50 repeats was associated with increased risk of corneal transplantation [12,13,14,15]. The effect of *TCF4* on FECD could be an alteration of the gene expression of specific TCF4 isoforms [8]. Furthermore, an oxidant-antioxidant imbalance was hypothesized [8,16,17].

Further studies have strengthened the association of *TCF4* single nucleotide polymorphisms in the FECD disease process, implicating the overexpression of CLU (Clusterin) and transforming growth factor, *β*-induced (TGFBI) [18]. CLU is a molecular chaperone, involved in the protection of cells from the effects of physiological stress due to aging and oxidative stress. TGFBI is an extracellular matrix protein that mediates cell adhesion [19], and FECD is characterized by a breakdown of tight junctions between corneal endothelial cells. In the Australian FECD cohort, immunohistochemistry showed differential expression of CLU and TGFBI proteins in FECD-affected compared with normal corneas [20].

DSO without the addition of Rho-associated protein kinase (ROCK) inhibitor has been reported in the literature as in intervention for Fuchs Dystrophy with varying levels of success, with corneal clearance usually taking between 3 and 6 months, with some cases failing to achieve clearance [1,2,3,4,5,6]. In view of the suggested contribution of *TCF4* sequence variants to the pathogenesis of FECD in terms of susceptibility to oxidative stress and cell adhesion, this is the first study to investigate whether the CTG expansion load contributes to time to resolution of corneal edema after DSO.

Limitations of the study include the small number of cases included and the lack of clear clinical phenotyping of cases pre-operatively.

It is possible that there may have been some selection bias, as patients meeting inclusion criteria for this study had mild FECD and good peripheral cell counts. Similarities in the phenotype may reflect some, as yet unknown common pathogenesis, which could be genetic, environmental, or most likely an interaction of the two, and may have an impact on response to surgery. Lastly, the surgery was carried out by multiple surgeons, and there is some evidence to suggest that surgical technique may have an impact on outcome in DSO [2,21].

## Figures and Tables

**Table 1:** Demographic details and pre-operative phenotypes, as well as time to clearance of the 11 eyes included in the study.

Eye	*TCF4* Repeats	Age	ECC (Superior)	CCT	Time to Clearance
1	11/27	56	2071	601	3 months
2	11/11	60	1518	616	3 months
3	11/62	71	2274	699	2 months
4	11/69	64	2041	629	3 months
5	63/73	69	2149	617	3 months
6	11/80	73	1785	651	Non-clearance
7	17/95	84	850	662	6 months
8	63/73	58	3146	589	6 months
9	11/123	78	Not recorded	602	Non-clearance
10	11/97	52	2470	534	2 months
11	14/110	39	2589	577	Non-clearance

**Table 2:** Results of prediction models for corneal clearance.

Explanatory Variable	VIP (SD) for Clearance Time	VIP (SD) for Clearance vs. No Clearance
Age	1.23 (1.32)	0.09 (0.95)
TCF4	0.83 (0.75)	1.52 (0.12)
ECC sup	0.41 (1.24)	0.23 (0.47)
CCT	0.26 (0.65)	0.23 (0.48)

VIP = variable importance for protection; SD = standard deviation; ECC = endothelial cell count; CCT = central corneal thickness.

## Data Availability

The data presented in this study are available on request from the corresponding author.

## References

[1] Arbelaez J.G., Price M.O., Price F.W. (2014). Long-term follow-up and complications of stripping descemet membrane without placement of graft in eyes with Fuchs endothelial dystrophy. Cornea.

[2] Koenig S.B. (2015). Planned Descemetorhexis Without Endothelial Keratoplasty in Eyes With Fuchs Corneal Endothelial Dystrophy. Cornea.

[3] Moloney G., Chan U.T., Hamilton A., Zahidin A.M., Grigg J.R., Devasahayam R.N. (2015). Descemetorhexis for Fuchs’ dystrophy. Can. J. Ophthalmol..

[4] Borkar D.S., Veldman P., Colby K.A. (2016). Treatment of Fuchs Endothelial Dystrophy by Descemet Stripping Without Endothelial Keratoplasty. Cornea.

[5] Moloney G., Petsoglou C., Ball M., Kerdraon Y., Hollhumer R., Spiteri N., Beheregaray S., Hampson J., D’Souza M., Devasahayam R.N. (2017). Descemetorhexis Without Grafting for Fuchs Endothelial Dystrophy-Supplementation With Topical Ripasudil. Cornea.

[6] Iovieno A., Neri A., Soldani A.M., Adani C., Fontana L. (2017). Descemetorhexis Without Graft Placement for the Treatment of Fuchs Endothelial Dystrophy: Preliminary Results and Review of the Literature. Cornea.

[7] Moloney G., Congote D.G., Hirnschall N., Arsiwalla T., Boso A.L.M., Toalster N., D’Souza M., Devasahayam R.N. (2021). Descemet Stripping Only Supplemented With Topical Ripasudil for Fuchs Endothelial Dystrophy 12-Month Outcomes of the Sydney Eye Hospital Study. Cornea.

[8] Wieben E.D., Aleff R.A., Tosakulwong N., Butz M.L., Highsmith W.E., Edwards A.O., Baratz K.H. (2012). A common trinucleotide repeat expansion within the transcription factor 4 (TCF4, E2-2) gene predicts Fuchs corneal dystrophy. PLoS ONE.

[9] Kuot A., Hewitt A.W., Snibson G.R., Souzeau E., Mills R., Craig J., Burdon K.P., Sharma S. (2017). TGC repeat expansion in the TCF4 gene increases the risk of Fuchs’ endothelial corneal dystrophy in Australian cases. PLoS ONE.

[10] Hirnschall N., Amir-Asgari S., Maedel S., Findl O. (2013). Predicting the postoperative intraocular lens position using continuous intraoperative optical coherence tomography measurements. Investig. Ophthalmol. Vis. Sci..

[11] Saade J.S., Xing C., Gong X., Zhou Z., Mootha V.V. (2018). Instability of TCF4 Triplet Repeat Expansion With Parent-Child Transmission in Fuchs’ Endothelial Corneal Dystrophy. Investig. Ophthalmol. Vis. Sci..

[12] Baratz K.H., Tosakulwong N., Ryu E., Brown W.L., Branham K., Chen W., Tran K.D., Schmid-Kubista K.E., Heckenlively J.R., Swaroop A. (2010). E2-2 protein and Fuchs’s corneal dystrophy. N. Engl. J. Med..

[13] Soliman A.Z., Xing C., Radwan S.H., Gong X., Mootha V.V. (2015). Correlation of Severity of Fuchs Endothelial Corneal Dystrophy With Triplet Repeat Expansion in TCF4. JAMA Ophthalmol..

[14] Eghrari A.O., Vasanth S., Wang J., Vahedi F., Riazuddin S.A., Gottsch J.D. (2017). CTG18.1 Expansion in TCF4 Increases Likelihood of Transplantation in Fuchs Corneal Dystrophy. Cornea.

[15] Zarouchlioti C., Sanchez-Pintado B., Tear N.J.H., Klein P., Liskova P., Dulla K., Semo M., Vugler A.A., Muthusamy K., Dudakova L. (2018). Antisense Therapy for a Common Corneal Dystrophy Ameliorates TCF4 Repeat Expansion-Mediated Toxicity. Am. J. Hum. Genet..

[16] Jurkunas U.V., Rawe I., Bitar M.S., Zhu C., Harris D.L., Colby K., Joyce N.C. (2008). Decreased expression of peroxiredoxins in Fuchs’ endothelial dystrophy. Investig. Ophthalmol. Vis. Sci..

[17] Jurkunas U.V., Bitar M.S., Funaki T., Azizi B. (2010). Evidence of oxidative stress in the pathogenesis of fuchs endothelial corneal dystrophy. Am. J. Pathol..

[18] Igo R.P., Kopplin L.J., Joseph P., Truitt B., Fondran J., Bardenstein D., Aldave A.J., Croasdale C.R., Price M.O., Rosenwasser M. (2012). Differing roles for TCF4 and COL8A2 in central corneal thickness and fuchs endothelial corneal dystrophy. PLoS ONE.

[19] Jurkunas U.V., Bitar M., Rawe I. (2009). Colocalization of increased transforming growth factor-beta-induced protein (TGFBIp) and Clusterin in Fuchs endothelial corneal dystrophy. Investig. Ophthalmol. Vis. Sci..

[20] Kuot A., Hewitt A.W., Griggs K., Klebe S., Mills R., Jhanji V., Craig J., Sharma S., Burdon K.P. (2012). Association of TCF4 and CLU polymorphisms with Fuchs’ endothelial dystrophy and implication of CLU and TGFBI proteins in the disease process. Eur. J. Hum. Genet..

[21] Davies E., Jurkunas U., Pineda R. (2018). Predictive Factors for Corneal Clearance After Descemetorhexis Without Endothelial Keratoplasty. Cornea.

